# Low urine pH affects the development of metabolic syndrome, associative with the increase of dyslipidemia and dysglycemia: Nationwide cross-sectional study (KNHANES 2013-2015) and a single-center retrospective cohort study

**DOI:** 10.1371/journal.pone.0202757

**Published:** 2018-08-24

**Authors:** Seung Min Chung, Jun Sung Moon, Ji Sung Yoon, Kyu Chang Won, Hyoung Woo Lee

**Affiliations:** Division of Endocrinology and Metabolism, Department of Internal Medicine, Yeungnam College of Medicine, Daegu, Republic of Korea; University of Colorado Denver School of Medicine, UNITED STATES

## Abstract

**Introduction:**

Low urine pH (UpH) and high serum uric acid are considered evidence of metabolic disorders. The effect of low UpH on the development of metabolic syndrome (MetS) is less clear than that of high serum uric acid. We investigated the association between low UpH on the development of MetS and its components: central obesity, dyslipidemia, hypertension, and dysglycemia.

**Methods:**

Two studies were conducted based on 2 datasets. The cross-sectional study included 14,511 subjects aged 19–80 years, based on the Korea National Health and Nutrition Examination Survey in 2013–2015. The retrospective cohort study included 3,453 subjects aged 19–80 years without MetS at the first checkup, who underwent at least 3 health checkups at a single tertiary hospital between 2011 and 2017. UpH was measured using an automatic urine analyzer in the range of 5.0–9.0 at first visit.

**Results:**

In the cross-sectional study, low UpH (= 5.0) was associated with the prevalence of MetS (odds ratio [OR] = 1.480, 95% confidence interval [CI] 1.334–1.643, p<0.001), particularly central obesity, dyslipidemia, and dysglycemia (OR ranges 1.282–1.422, p<0.001, all). In the retrospective cohort study, compared with the highest UpH subgroup, the lowest UpH subgroup (= 5.0) was associated with higher risk of MetS development (hazard ratio = 1.394, 95% CI 1.096–1.772, p = 0.007). The incident risk of MetS increased from the highest to lowest UpH subgroups (p for trend = 0.020), among which dyslipidemia and dysglycemia increased (p for trend <0.01, all).

**Conclusion:**

Low UpH can be used as a surrogate marker of MetS and affects the development of MetS, associative with the increase of dyslipidemia and dysglycemia in those without MetS. If UpH is ≤5.0, efforts to prevent metabolic disorders are warranted.

## Introduction

Metabolic syndrome (MetS) includes central obesity, dyslipidemia, hypertension, and dysglycemia components [1]. The more prominent the components of MetS, the higher the incidence of type 2 diabetes and cardiovascular mortality [2, 3]. In recent decades, marked environmental and lifestyle changes have occurred in Asia, including Korea, leading to a rapid increase in the incidence of MetS.

In patients with MetS, uric acid nephrolithiasis is significantly more common [4, 5], and two mechanisms have been suggested; 1) increased acid excretion due to dietary habits (increased intake of animal protein and salt, decreased intake of alkaline-rich fruits and vegetables); 2) decreased renal ammoniagenesis and ammonium excretion due to increased insulin resistance [6–8]. Both mechanisms eventually decrease urine pH (UpH) and increase insoluble uric acid, ultimately increasing uric acid nephrolithiasis, which can be considered evidence of a metabolic disorder [4].

Various cohort studies have proven that an increase in serum uric acid (SUA) increased the development of metabolic syndrome [9–13], hypertension [14], type 2 diabetes [15, 16], stroke [17], and cardiovascular mortality [18]. However, a causal relationship between UpH and MetS is unclear, and most studies have had a cross-sectional design [19–22]. UpH is an easy test, so it can be used as an early predictor if the association between low UpH and development of MetS is proven. Accordingly, we aimed to validate the relationship between UpH and the prevalence of MetS using a cross-sectional study, and investigate the effect of low UpH on the development of MetS and its components through a retrospective longitudinal cohort study.

## Subjects and methods

### Study participants

We conducted cross-sectional and retrospective longitudinal cohort studies based on 2 datasets.

**Fig 1:**
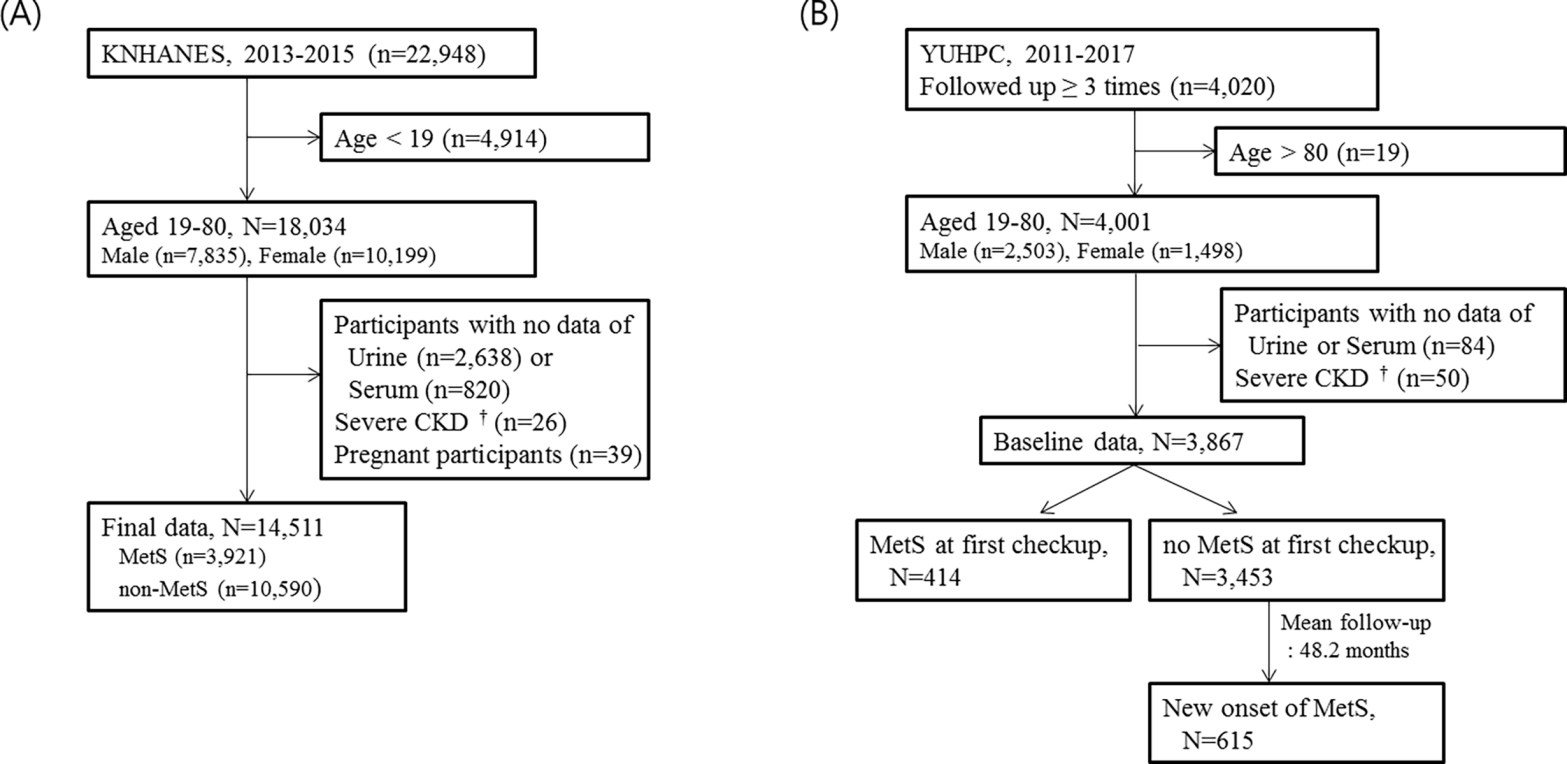
Description of study population. (A) Cross-sectional study based on KNHANES 2013–2015 and (B) retrospective longitudinal cohort study based on YUHPC. †estimated glomerular filtration rate < 30 ml/min/1.73 m^2^.

The first study was the cross-sectional study based on the data of the Korea National Health and Nutrition Examination Survey (KNHANES) in 2013–2015. KNHANES is a cross-sectional, population-based, nationwide survey that is regularly conducted by the Korea Centers for Disease Control and Prevention [23]. Of the 22,948 participants in the 2013–2015 KNHANES, we initially selected individuals aged 19–80 years old (total 18,034: 7,835 males and 10,199 females). Exclusion criteria were as follows: participants without urine data (n = 2,638) or serum data (n = 820); participants who had severe chronic kidney disease (estimated glomerular filtration rate [eGFR] <30 mL/min/1.73 m^2^, n = 26); and participants who were pregnant (n = 39). A total of 14,511 subjects were included in the final analysis (Fig 1A). All participants gave written informed consent. The survey protocol was approved by the institutional review board of the Korean Centers for Disease Control and Prevention.

The second study was the retrospective longitudinal cohort study based on the data of the Yeungnam University Hospital Health Promotion Center (YUHPC) (Daegu, South Korea). Of the 4,020 participants who received a health checkup at least 3 times between 2011 and 2017, we selected individuals aged 19–80 years old (total 4,001: 2,503 males and 1,498 females). Participants without urine or serum data (n = 84) and participants who had severe chronic kidney disease (eGFR <30 ml/min/1.73 m^2^, n = 50) at first checkup were excluded. A total of 3,867 subjects were included in the baseline analysis. Participants received a health checkup at intervals of 6 months to 3 years. The mean follow-up period was 48.2 ± 17.5 months (range 6–83). The new onset of MetS during follow-up was analyzed among subjects without MetS at first checkup (total 3,453: 2,130 males and 1,323 females) (Fig 1B). All participants gave written informed consent. The study was approved by the institutional review board of Yeungnam University Hospital (YUMC 2018-02-011).

### Measurements of biochemical and clinical variables

KNHANES data for sociodemographic characteristics, lifestyle factors, nutritional status, medical history, anthropometric values, and blood and urine tests were examined. Educational status was classified as less than high school graduation or more than high school graduation. Income status was classified by the income-based poverty rate, which was divided into quartiles; the first and second quantiles were defined as low income, and the third and fourth quantiles were defined as high income. Smoking status was classified as non- or ex-smoker and current-smoker (≥100 cigarettes so far), and drinking status was classified as non-drinker and current-drinker (≥1 drink per month in the prior year). Regular exercise was defined as moderate-intensity physical activity (≥2 h 30 min/week), or high-intensity physical activity (≥1 h 15min/week), or combined moderate and high intensity physical activity (1 min at high intensity = 2 min at moderate intensity). Food intake status was classified as stable or unstable by grouping the summation of 18 questions based on the US household food security/hunger survey module. Daily energy and nutrient intake were calculated using a food intake frequency survey, which the questionnaire consisted of 112 food items; the intake frequency of the items was divided into 9 responses, and the amount of intake was composed of 3 responses [24]. Blood pressure (BP) was calculated as the average of second and third BP readings. Height, weight, and waist circumference (WC) were measured by trained staff members. Body mass index (BMI) was calculated as body weight in kilograms divided by the height in meters squared (kg/m^2^). Blood samples and spot urine samples were collected after 8 h fasting. The homeostasis model assessment of insulin resistance (HOMA-IR) and beta-cell function (HOMA-B) were calculated with the following formulas: HOMA-IR = Glucose × Insulin /405 and HOMA-B = 360 × Insulin /(Glucose– 63) [25]. The eGFR was calculated using the Modification of Diet in Renal Disease Study formula: 186 × (creatinine)^–1.154^ × (age)^–0.203^ × (0.742 if female) × (1.210 if black). UpH was measured using a Urisys 2400 analyzer (Roche, Germany) and was categorized into the following values: 5.0, 6.0, 6.5, 7.0, 8.0, and 9.0.

Data of the YUHPC included anthropometric values and blood and urine test results. BMI was calculated as described above. Seated BP was measured twice and a mean value was calculated. All laboratory tests were performed in the central laboratory of Yeungnam University Hospital. Antecubital venous sampling was performed after an overnight fast. HOMA-IR, HOMA-B, and eGFR were calculated as described above, and low-density lipoprotein cholesterol (LDL-cho) was calculated as: Total cholesterol (T-cho)–High-density lipoprotein cholesterol (HDL-cho)–Triglyceride (TG)/5. The SUA and eGFR values were measured as adjustment factors when assessing the impact of UpH on development of MetS. Midstream urine samples were collected during the morning after an overnight fast. UpH was measured at the first time point using a URiSCAN Super analyzer (YD Diagnostics, Korea) and was categorized into the following values: 5.0, 5.5, 6.0, 6.5, 7.0, 7.5, 8.0, and 8.5.

### Definition of MetS and its components

MetS was defined according to the joint interim statement of the International Diabetes Federation criteria and National Cholesterol Education Program-Adult Treatment Panel III criteria [26]. A diagnosis of MetS required 3 of the following 5 factors: central obesity, with waist circumference (WC) ≥90 cm for Korean men and ≥85 cm for Korean women [27]; raised-TG, with TG ≥150 mg/dL (1.7 mmol/L) or specific treatment for this lipid abnormality; reduced-HDL, with HDL-Cho <40 mg/dL (1.0 mmol/L) in males and <50 mg/dL (1.3 mmol/L) in females or specific treatment for this lipid abnormality; raised-BP, with systolic BP (SBP) ≥130 or diastolic BP (DBP) ≥85 mmHg or treatment of previously diagnosed hypertension; and raised fasting plasma glucose (FPG), with FPG ≥100 mg/dL (5.6 mmol/L) or previously diagnosed type 2 diabetes.

### Statistical analysis

All statistical analyses were performed using SPSS (version 21.0, IBM Inc., Chicago, IL, USA). In comparing the features of MetS and non-MetS, baseline characteristics were presented as mean ± standard deviation (SD) values for continuous variables and as frequencies with percentages for categorical variables. The statistical significance of differences in continuous variables and categorical variables between 2 groups was determined using an independent sample T-test and Pearson’s chi-square test. We defined the low UpH as < 5.5, with reference to previous studies [20, 21]. P values of <0.05 were considered statistically significant. In the multiple logistic regression analysis, the odds ratios (ORs) of UpH for MetS was analyzed by dividing UpH = 5.0 and, > 6.0. In the Cox regression analysis, the hazard ratios (HRs) of UpH for MetS was analyzed by subdividing UpH into = 5.0, = 5.5, 6.0–6.5, and, ≥7.0. ORs and HRs were reported with their 95% confidence intervals (95% CIs). For multiple testing, P values adjusted by Benjamini-Hochberg procedure were considered statistically significant to reduce the false discovery rate (S1 Table) [28].

## Results

### Nationwide cross-sectional study (KNHANES, 2013–2015)

The mean age was 50.6 ± 16.1 years (range 19–80) and 44.9% were males. The prevalence of MetS, central obesity, raised-TG, reduced-HDL, raised-BP, and raised-FPG was 27.0%, 26.3%, 35.1%, 33.3%, 39.4% and 31.8%, respectively. The values of each MetS components are presented in Table 1. The proportion of participants with UpH 5.0, 6.0–6.5, and ≥7.0 was 51.8%, 31.6%, and 16.6%, respectively. Table 2 compares demographic and metabolic characteristics of 14,511 participants in MetS and non-MetS groups (n = 3,921 vs. n = 10,590). The MetS group was significantly older, male predominant, less educated, and had lower income. In the MetS group, the percentage of current smokers was significantly higher, the percentage of current drinkers and regular exercisers was significantly lower, and food intake stability, daily total caloric intake, and protein and fat intake were significantly lower. Carbohydrate intake was not statistically different between groups. In the MetS group, systolic and diastolic BP, WC, BMI, FPG, HbA1c, fasting insulin, HOMA-IR, HOMA-B, T-cho, and TG values were significantly higher, and HDL-cho was significantly lower. LDL-cho was not significantly different between the groups. In the MetS group, the proportion with UpH = 5.0 compared to those with UpH ≥6.0 was higher (56.4% vs. 50.1%, p<0.001).

**Table 1:** Prevalence and values of MetS and its components.

	Prevalence, n (%)	Values, Mean (min, max)	
**MetS**	3,921 (27.0)		
**Central Obesity**	3,813 (26.3)	WC, cm	93.7 (63, 130)
		BMI, kg/m2	27.6 (19, 49)
**Raised-TG**	5,096 (35.1)	TG, mmol/L [mg/dL]	2.55 (0.36, 21.1) [226.0 (32, 1868)]
**Reduced-HDL**	4,839 (33.3)	HDL, mmol/L [mg/dL]	1.09 (0.52, 2.64) [42.1 (20, 102)]
**Raised-BP**	5,712 (39.4)	SBP, mmHg	132.6 (85, 219)
		DBP, mmHg	81.1 (34, 138)
**Raised-FPG**	4,618 (31.8)	FPG, mmol/L [mg/dL]	6.63 (2.66, 22.48) [119.5 (48, 405)]

**Table 2:** Demographics and metabolic characteristics of KHANES (2013–2015) participants.

	Total (n = 14,511)	Non-MetS (n = 10,590)	MetS (n = 3,921)	P-value
Age, y	50.6 ± 16.1	47.6 ± 16.0	58.7 ± 13.4	<0.001
Male gender, n (%)	6,515 (44.9)	4,616 (43.6)	1,899 (48.4)	<0.001
Less than high school education, n (%)^1^	4,508 (33.3)	2,523 (26.2)	1,985 (51.2)	<0.001
Low income, n (%)^2^	7,074 (49.0)	5,070 (48.2)	2,004 (51.3)	0.001
**Lifestyle patterns**				
Current smoker, n (%)^3^	5,263 (36.3)	3,614 (36.1)	1,649 (42.6)	<0.001
Current drinker, n (%)^4^	7,547 (54.3)	5,589 (55.7)	1,958 (50.5)	<0.001
Regular exercise, n (%)^5^	4,498 (50.6)	3,358 (53.5)	1,140 (43.5)	<0.001
**Dietary intake**				
Food intake stability, n(%)^6^	12,275 (91.9)	8,984 (92.3)	3,291 (90.9)	0.009
Energy intake, Kcal/d	2019.4 ± 926.1	2046.9 ± 933.3	1946.4 ± 902.4	<0.001
Protein Intake, g/d	69.4 ± 44.6	71.0 ± 46.5	65.2 ± 38.8	<0.001
Fat intake, g/d	42.5 ± 35.5	45.1 ± 36.6	35.7 ± 31.3	<0.001
Carbohydrate intake, g/d	314.0 ± 129.5	314.2 ± 130.8	313.5 ± 126.0	0.782
**Body measurement**				
SBP, mmHg	118.4 ± 16.8	114.8 ± 15.5	128.1 ± 16.1	<0.001
DBP, mmHg	75.1 ± 10.3	73.7 ± 9.7	78.8 ± 11.2	<0.001
WC, cm	81.7 ± 9.9	78.8 ± 8.7	89.3 ± 8.6	<0.001
BMI, kg/m^2^	23.9 ± 3.4	23.0 ± 3.0	26.2 ± 3.4	<0.001
**Biochemistry**				
Fasting Glucose, mmol/L [mg/dL]	5.6 ± 1.3 [100.5 ± 23.7]	5.3 ± 1.1 [95.8 ± 20.0]	6.3 ± 1.6 [113.1 ± 28.1]	<0.001
HbA1c, %	5.8 ± 0.8	5.6 ± 0.6	6.2 ± 1.0	<0.001
Fasting Insulin, uU/mL	8.6 ± 8.3	7.5 ± 7.5	11.1 ± 9.4	<0.001
HOMA-IR	2.3 ± 3.1	1.9 ± 3.0	3.2 ± 3.3	<0.001
HOMA-B	87.6 ± 89.0	85.2 ± 91.9	93.1 ± 81.7	0.004
T-Cho, mmol/L [mg/dL]	4.89 ± 0.92 [188.9 ± 35.5]	4.87 ± 0.88 [188.1 ± 34.0]	4.95 ± 1.02 [191.3 ± 39.2]	<0.001
TG, mmol/L [mg/dL]	1.54 ± 1.22 [136.6 ± 108.4]	1.26 ± 0.90 [111.5 ± 79.7]	2.31 ± 1.60 [204.2 ± 141.6]	<0.001
HDL-Cho, mmol/L [mg/dL]	1.32 ± 0.32 [50.9 ± 12.2]	1.39 ± 0.31 [53.7 ± 11.8]	1.13 ± 0.25 [43.6 ± 9.8]	<0.001
LDL-Cho, mmol/L [mg/dL]	2.95 ± 0.84 [114.0 ± 32.6]	2.96 ± 0.82 [114.4 ± 31.6]	2.94 ± 0.89 [113.4 ± 34.3]	0.233
**Urine pH**				
UpH ≥ 6.0, n(%)	6,996 (48.2)	5,286 (49.9)	1,710 (43.6)	<0.001
UpH = 5.0, n(%)	7,515 (51.8)	5,304 (50.1)	2,211 (56.4)	

Data are presented as mean ± SD or frequencies with percentage.

Subjects who had missing data

^1^ 988

^2^ 86

^3^ 621

^4^ 605

^5^ 5,613, and

^6^ 1,159.

The ORs of UpH for MetS and its components were analyzed using multiple logistic regression analysis (Table 3). UpH was analyzed as a continuous covariate and categorical covariates were divided into UpH ≥6.0 (reference range) and UpH = 5.0. The logistic regression model was adjusted for age, sex, educational status, income, smoking, drinking, exercise, and daily intake of protein, fat, and carbohydrate. After adjustment, the ORs for MetS, central obesity, raised-TG, reduced-HDL, and raised-FPG were significantly increased, with UpH analyzed using both continuous (OR ranges 1.163–1.310, all p<0.001) and categorical (OR ranges 1.282–1.480, all p<0.001) covariates. The ORs for raised-BP were not statistically significant for either continuous or categorical UpH variables after adjustment.

**Table 3:** Multiple logistic regression analysis for metabolic syndrome and its components according to the value of UpH.

	Crude OR (95% CI)			Adjusted OR ^†^ (95% CI)		
	UpH, as the value decreases	UpH≥6.0	UpH = 5.0	UpH, as the value decreases	UpH≥6.0	UpH = 5.0
MetS	1.160*** (1.110–1.212)	1 (Ref)	1.289*** (1.197–1.387)	1.310*** (1.232–1.394)	1 (Ref)	1.480*** (1.334–1.643)
Central Obesity	1.134*** (1.085–1.186)	1 (Ref)	1.229*** (1.141–1.324)	1.214*** (1.143–1.290)	1 (Ref)	1.313*** (1.187–1.453)
Raised-TG	1.138*** (1.093–1.185)	1 (Ref)	1.276*** (1.192–1.367)	1.163*** (1.098–1.231)	1 (Ref)	1.282*** (1.164–1.412)
Reduced-HDL	1.101*** (1.057–1.147)	1 (Ref)	1.223*** (1.141–1.311)	1.183*** (1.116–1.253)	1 (Ref)	1.342*** (1.218–1.479)
Raised-BP	0.931*** (0.896–0.968)	1 (Ref)	0.922* (0.863–0.986)	1.039 (0.978–1.103)	1 (Ref)	1.004 (0.906–1.113)
Raised-FPG	1.143*** (1.095–1.095)	1 (Ref)	1.261*** (1.173–1.354)	1.252*** (1.180–1.330)	1 (Ref)	1.422*** (1.286–1.573)

***p<0.001

**p<0.01

*p<0.05

^†^ Adjusted for age, sex, educational status, income, smoking, drinking, exercise, and daily intake of protein, fat and carbohydrate.

OR, odds ratio; CI, confidence interval

### Retrospective cohort study (YUHPC, 2011–2017)

The demographic and metabolic characteristics of 3,867 participants at first checkup are shown in Table 4. The mean age was 52.7 ± 9.8 years (range 19–80) and 63.6% were males. The prevalence of MetS, central obesity, raised-TG, reduced-HDL, raised-BP, and raised-FPG was 10.7%, 15.1%, 27.4%, 19.5%, 22.0%, and 16.9%, respectively. The proportion of participants with UpH = 5.0, = 5.5, 6.0–6.5, and, ≥7.0 was 53.0%, 12.7%, 18.3%, and 16.1%, respectively. Compared to the non-MetS group (n = 3,453), the MetS group (n = 414) was significantly older and male predominant, with higher systolic BP, diastolic BP, WC, BMI, FPG, HbA1c, fasting insulin, HOMA-IR, HOMA-B, T-cho, and TG values, and lower HDL-cho values. LDL-cho was not significantly different between the groups. The mean SUA value was significantly higher (6.0 ± 1.4 vs. 5.2 ± 1.4, p<0.001) in the MetS group. The proportions with UpH = 5.0, = 5.5, 6.0–6.5, or ≥7.0 were significantly different; the proportion of subgroups with lower UpH values was higher in the MetS group (p for trend <0.001).

**Table 4:** Demographics and metabolic characteristics of YUHPC (2011–2017) participants at first checkup.

	Total (n = 3,867)	Non-MetS (n = 3,453)	MetS (n = 414)	P-value
Age, y	52.7 ± 9.8	52.5 ± 9.9	54.2 ± 9.5	<0.001
Male gender, n (%)	2,458 (63.6)	2,130 (61.7)	328 (79.2)	<0.001
Central obesity, n(%)	585 (15.1)	329 (9.5)	256 (61.8)	<0.001
Raised-TG, n(%)	1,058 (27.4)	705 (20.4)	353 (85.3)	<0.001
Reduced-HDL, n(%)	754 (19.5)	500 (14.5)	254 (61.4)	<0.001
Raised-BP, n(%)	852 (22.0)	588 (17.0)	264 (63.8)	<0.001
Raised-FPG, n(%)	653 (16.9)	402 (11.6)	251 (60.6)	<0.001
SBP, mmHg	120.7 ± 113.5	119.2 ± 12.9	132.6 ± 13.1	<0.001
DBP, mmHg	76.1 ± 10.0	75.1 ± 9.5	84.7 ± 9.6	<0.001
WC, cm	80.4 ± 8.2	79.3 ± 7.7	89.3 ± 7.0	<0.001
BMI, kg/m^2^	23.7 ± 2.9	23.3 ± 2.7	26.8 ± 2.8	<0.001
Fasting Glucose, mmol/L [mg/dL]	5.2 ± 1.1 [93.0 ± 19.3]	5.1 ± 0.9 [91.0 ± 15.8]	6.1 ± 1.8 [110.0 ± 32.5]	<0.001
HbA1c, %	5.6 ± 0.7	5.6 ± 0.6	6.2 ± 1.2	<0.001
Fasting Insulin, uU/mL	6.0 ± 3.6	5.5 ± 3.3	9.6 ± 3.7	<0.001
HOMA-IR	1.5 ± 1.1	1.3 ± 0.9	2.6 ± 1.5	<0.001
HOMA-B	73.4 ± 50.3	70.6 ± 50.9	91.3 ± 42.9	0.002
T-Cho, mmol/L [mg/dL]	5.14 ± 0.90 [198.4 ± 34.7]	5.11 ± 0.89 [197.4 ± 34.2]	5.34 ± 0.99 [206.1 ± 38.2]	<0.001
TG, mmol/L [mg/dL]	1.45 ± 1.09 [128.4 ± 96.4]	1.30 ± 0.93 [115.4 ± 82.5]	2.68 ± 1.47 [236.8 ± 130.3]	<0.001
HDL-Cho, mmol/L [mg/dL]	1.42 ± 0.36 [54.9 ± 14.0]	1.46 ± 0.35 [56.3 ± 13.7]	1.11 ± 0.26 [43.0 ± 9.9]	<0.001
LDL-Cho, mmol/L [mg/dL]	3.05 ± 0.86 [117.8 ± 33.3]	3.06 ± 0.84 [118.0 ± 32.6]	3.00 ± 1.00 [115.8 ± 38.5]	0.192
Uric acid, mmol/L [mg/dL]	315.8 ± 85.1 [5.31 ± 1.43]	309.3 ± 83.3 [5.2 ± 1.4]	356.9 ± 83.3 [6.0 ± 1.4]	<0.001
UpH ≥ 7.0, n(%)	619 (16.1)	581 (16.8)	38 (9.2)	<0.001
UpH 6.0–6.5, n(%)	709 (18.3)	648 (18.8)	61 (14.7)	
UpH = 5.5, n(%)	490 (12.7)	419 (12.1)	71 (17.1)	
UpH = 5.0, n(%)	2,049 (53.0)	1,805 (52.3)	244 (58.9)	

Data are presented as mean ± SD or frequencies with percentage.

**Fig 2:**
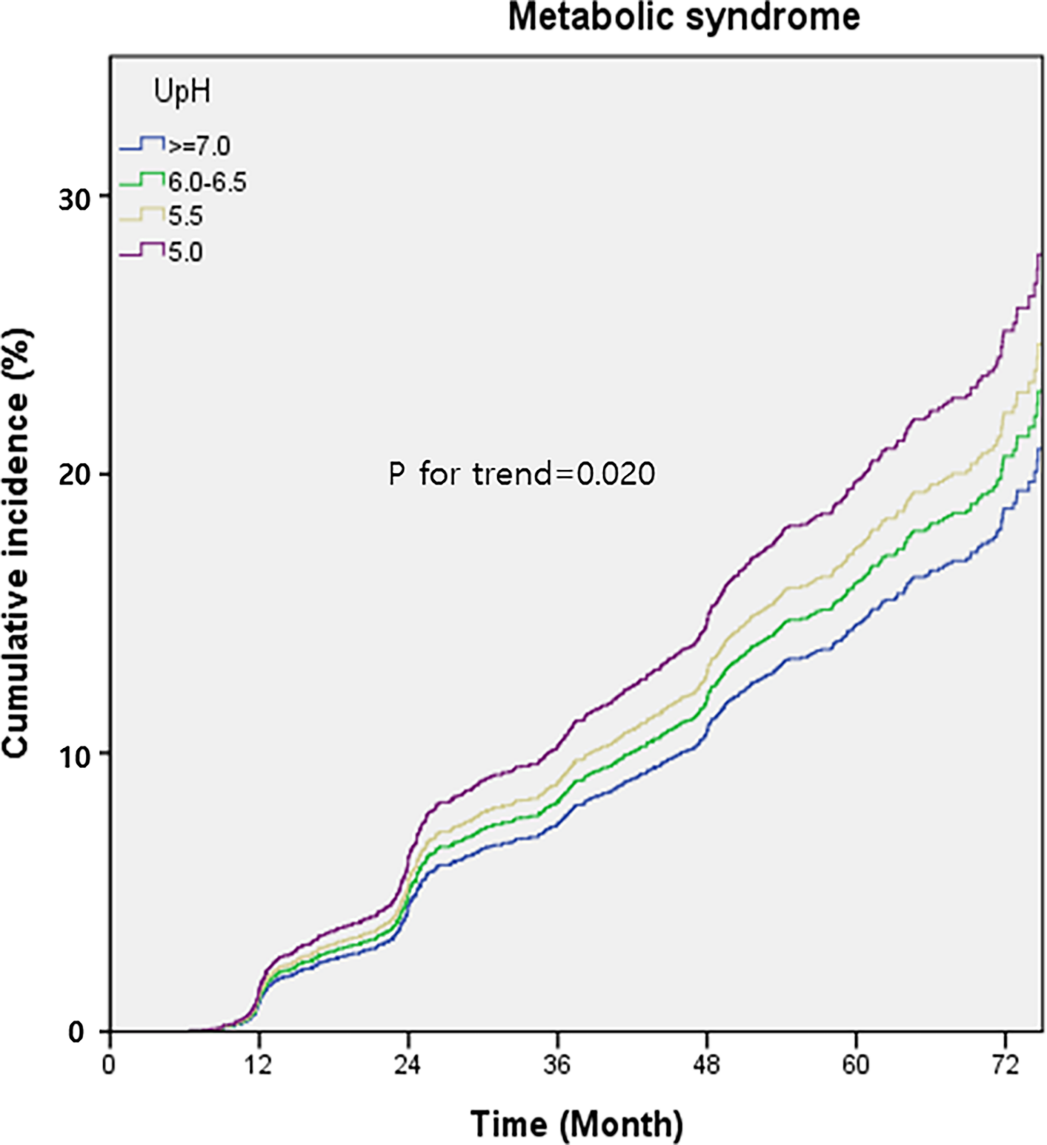
Cumulative incidences (%) of metabolic syndrome according to 4 UpH subgroups at first checkup. The cumulative incidence was calculated based on cox regression analysis adjusted for age, gender, eGFR, and serum uric acid at the first checkup. UpH, urine pH.

A total of 3,453 participants without MetS at first checkup were divided into 4 subgroups according to UpH values at first checkup: ≥7.0 (reference range), 6.0–6.5, = 5.5, and = 5.0. HRs for new onset of MetS and its components were analyzed using Cox regression analysis (Table 5). Within average follow-up period of 48.2 months (range 6–83), MetS was developed in 494 males and 141 females (total 635, 18.4%). The Cox regression model was adjusted for age, sex, eGFR, and SUA at the first checkup. After adjustment, the HRs of MetS, raised-TG, and raised-FPG increased significantly in the UpH = 5.0 subgroup compared to those in the reference subgroup (HR range 1.316–1.394, all p<0.01). The trend for incidence risk of MetS, raised-TG, reduced-HDL, and raised-FPG increased from the highest to lowest UpH subgroups (p for trend = 0.020, <0.001, 0.007, and 0.001, respectively). The HRs and trends for incidence risk of central obesity and raised-BP were not statistically significant. The cumulative incidence of MetS according to the 4 UpH subgroups is presented in Fig 2. Additional adjustments were made to raised-TG and raised-FPG to ensure that the association between UpH and one MetS component was not confused by other components (S2 Table). In addition to the adjustment factors in Table 5, the values of the other four MetS components were adjusted; WC, BMI, HDL-cho, SBP, DBP, and FPG (for raised-TG) or TG (for raised-FPG). Even after adjustment, the HRs of raised-TG (HR = 1.251, p<0.05) and raised-FPG (HR = 1.354, p<0.01) increased in the UpH = 5.0 subgroup compared to those in the reference group, and the trend for incidence risk also increased from the highest to lowest UpH subgroups (p for trend = 0.001 and 0.002, respectively).

**Table 5:** Cox regression analysis for new onset of MetS and its components, according to UpH values of 3,453 participants without MetS at first checkup.

	UpH≥7.0 (n = 581)	UpH 6.0–6.5 (n = 648)	UpH = 5.5 (n = 419)	UpH = 5.0 (n = 1,805)	P for trend
**Metabolic syndrome**					
Incidence (%)	14.6	18.2	18.7	19.6	
Crude HR (95% CI)	1 (ref)	1.219 (0.912–1.613)	1.265 (0.929–1.724)	1.455 (1.147–1.846) **	0.014
Adjusted HR^†^(95% CI)	1 (ref)	1.113 (0.841–1.473)	1.208 (0.886–1.647)	1.394 (1.096–1.772) **	0.020
**Central obesity**					
Incidence (%)	17.2	17.8	20.1	16.3	
Crude HR (95% CI)	1 (ref)	0.961 (0.735–1.257)	1.140 (0.853–1.524)	1.071 (0.854–1.344)	0.612
Adjusted HR^†^(95% CI)	1 (ref)	0.855 (0.653–1.119)	1.100 (0.823–1.472)	1.023 (0.814–1.287)	0.296
**Raised-TG**					
Incidence (%)	25.4	27.6	30.9	33.8	
Crude HR (95% CI)	1 (ref)	1.023 (0.823–1.273)	1.186 (0.936–1.504)	1.477 (1.234–1.769)***	<0.001
Adjusted HR^†^(95% CI)	1 (ref)	0.938 (0.754–1.167)	1.084 (0.855–1.375)	1.316 (1.097–1.578) **	<0.001
**Reduced-HDL**					
Incidence (%)	13.6	12.2	13.5	15.4	
Crude HR (95% CI)	1 (ref)	0.843 (0.617–1.152)	0.973 (0.691–1.370)	1.234 (0.961–1.585)	0.013
Adjusted HR^†^(95% CI)	1 (ref)	0.843 (0.617–1.152)	1.007 (0.714–1.420)	1.267 (0.984–1.633)	0.007
**Raised-BP**					
Incidence (%)	31.4	28.7	31.6	28.5	
Crude HR (95% CI)	1 (ref)	0.855 (0.697–1.049)	0.980 (0.783–1.227)	1.017 (0.858–1.204)	0.239
Adjusted HR^†^(95% CI)	1 (ref)	0.811 (0.660–0.995) *	0.948 (0.757–1.188)	0.975 (0.822–1.157)	0.143
**Raised-FPG**					
Incidence (%)	22.3	26.3	23.9	27.5	
Crude HR (95% CI)	1 (ref)	1.117 (0.889–1.404)	1.040 (0.800–1.352)	1.380 (1.137–1.675) **	0.001
Adjusted HR^†^(95% CI)	1 (ref)	1.074 (0.8454–1.351)	1.027 (0.790–1.336)	1.367 (1.124–1.662) **	0.001

***p<0.001

**p<0.01

*p<0.05

^**†**^ **A**djusted for age, sex, eGFR and serum uric acid at the first checkup

HR, hazard ratio; CI, confidence interval

## Discussion

Our results showed that low UpH (= 5.0) was associated with the prevalence of MetS, particularly central obesity, dyslipidemia (raised-TG, reduced-HDL), and dysglycemia (raised-FPG), independent of age, sex, educational status, income, smoking, drinking, exercise, and daily dietary intake. Furthermore, low UpH (= 5.0) was associated with the development of MetS, among which dyslipidemia and dysglycemia increased, independent of age, sex, eGFR, and SUA.

There are few studies on the relationship between UpH and MetS. A study based on the KNHANES 2010 showed that UpH <5.5 was significantly associated with MetS, particularly raised-TG, and raised-FPG [20]. A study from a single health care center in Japan showed an association between lower UpH and MetS and its components, except for central obesity [21] or raised-BP [22]. In a 5-year, retrospective, Japanese cohort study, the risk of incident MetS was significantly elevated in males with UpH ≤5.0, and a high SUA and low UpH had a synergistic effect on development of MetS [29]. Our results reemphasize previous study results: an UpH value of ≤5.0 affects the prevalence and development of MetS. Both previous studies and the present study measured fasting spot UpH instead of 24-h UpH. Fasting spot UpH is not only convenient, cost-effective, but also has a significant correlation with the 24-h UpH [30], and is suitable for health checkups or large-scale health behavior surveys.

In our study results, the prevalence and development of raised-BP was not significantly associated with UpH. UpH may not be associated with all MetS components. MetS is featured by changes in insulin resistance in different organs of the body, a concept designed to predict the risk of cardiovascular dysfunction and type 2 diabetes [26]. The insulin resistance in adipose tissue, highly correlated with central obesity, results in the inhibition of the conversion of lipid from carbohydrates for storage, which confuses lipid and glucose homeostasis and induces fatty organs. Insulin resistance in vascular endothelium promotes hypertension, atherosclerosis, and disrupts systemic insulin sensitivity and glucose homeostasis [31]. That is, raised-BP is associated with metabolic abnormalities, but is also a separate pathogenesis. Changes in UpH probably originate directly from the metabolic abnormalities; central obesity, dyslipidemia, and dysglycemia, but not hypertension.

We think that low UpH is a consequence of acidification of body fluids, and acidification of body fluids is the consequence of metabolic changes. An excessively low UpH is considered a renal manifestation of insulin resistance [4], which is associated with a decreased ratio of ammonium to net acid excretion. Impaired ammonium production and excretion are caused by overload of free fatty acids in relation to the obesity and obesity-related MetS [8, 32], and increased acid production is mainly due to obesity, MetS and type 2 diabetes [5]. Low UpH is not only the manifestation MetS, but also a factor that changes with metabolic changes, so it can be used as a predictor of progress in a person without MetS.

In our cross-sectional study, the daily intake of total energy, protein, and fat was significantly higher in the non-MetS group. Despite the many advantages of food intake frequency surveys, this may have been due to its limitations such as measurement error and recall error [33]; participants with MetS might have reported less than they actually ate or might already have started dietary adjustments. Effective changes in dietary habits (dietary approaches to stop hypertension) are required to reduce the risk of MetS [34] and alkalinize UpH [35].

We adjusted eGFR and SUA as potential confounding factors while evaluating the impact of UpH on MetS development. Progressive decline in eGFR decreases renal ammonium excretion, causing metabolic acidosis that may alter UpH [36]. Moreover, increased SUA is a proven predictor of MetS development and should be considered as a confounder of UpH in MetS development [37]. Our study revealed that low UpH significantly affects MetS development, even after adjustment for these confounding factors.

Our study had some limitations. First, SUA values were not collected in KNHANES data, and variables of medical history, smoking, drinking, exercise, and dietary habits were not collected in the YUHPC data. Second, in KNHANES data, the UpH value was less subdivided and there was no UpH value of 5.5. Third, our study was based on a single determination of UpH, which may lead to random measurement error. Fourth, the participants in the YUHPC did not reflect a nationwide population, so a larger cohort study is needed to empower our research results. Finally, since these studies are mainly conducted in Asia, there may be differences depending on race, so further research is needed on other races.

Despite these limitations, our findings have the strength of a nationwide cross-sectional study and a relatively large, longitudinal cohort study. This is the first cohort study to analyze the contribution of UpH to development of MetS and its specific components. As our study showed that low UpH is significantly associated with the increase of dyslipidemia and dysglycemia, further study of the relationship between changes in UpH and beta-cell dysfunction, or progression of type 2 diabetes is essential.

In conclusion, low UpH can be used as a surrogate marker of MetS and affects the development of MetS, associative with the increase of dyslipidemia and dysglycemia in those without MetS. If UpH is ≤5.0, efforts to prevent metabolic disorders are warranted.

## Supporting information

S1 Table Adjusted p-values for multiple testing (corresponds to Tables 3 and 5) using Benjamini-Hochberg procedure. n, number of tests performed; d, maximum false discovery rate (often 0.05). 1. Sort the P-values in ascending order label as P_1_, P_2_,., P_n_. 2. P_i_ values of < d x i/n are considered statistically significant.
(DOCX)

S2 Table Additional cox regression analysis for new onset of Raised-TG and Raised-FPG, according to UpH values of participants without MetS at first checkup. Raised-TG: Adjusted for age, sex, eGFR, serum uric acid, and the values of another four MetS components; WC, BMI, HDL-cho, SBP, DBP, and FPG. Raised-FPG: Adjusted for age, sex, eGFR, serum uric acid, and the values of another four MetS components; WC, BMI, HDL-cho, SBP, DBP, and TG.
(DOCX)
