# Supplementary material for: Low urine pH affects the development of metabolic syndrome, associative with the increase of dyslipidemia and dysglycemia: Nationwide cross-sectional study (KNHANES 2013-2015) and a single-center retrospective cohort study
Source: PLoS One. 2018 Aug 24;13(8):e0202757. doi: 10.1371/journal.pone.0202757 (PMC6108487; doi:10.1371/journal.pone.0202757)
Supplement: S1 Table — n, number of tests performed; d, maximum false discovery rate (often 0.05). 1. Sort the P-values in ascending order label as P1, P2,., Pn. 2. Pi values of < d x i/n are considered statistically significant. (DOCX) [file pone.0202757.s001.docx]

**S1 Table. Adjusted p-values for multiple testing (corresponds to Tables 3 and 5) using Benjamini-Hochberg procedure.**

| Sorted P-values (P_i_) | P_1_ | P_2_ | P_3_ | P_4_ | P_5_ | P_6_ |
| --- | --- | --- | --- | --- | --- | --- |
| Adjusted p-values of significance (d x i/n) | 0.008 | 0.017 | 0.025 | 0.033 | 0.042 | 0.05 |

n, number of tests performed; d, maximum false discovery rate (often 0.05)

1. Sort the P-values in ascending order label as P_1_, P_2_, ., P_n_.

2. P_i_ values of < d x i/n are considered statistically significant.
