# Supplementary material for: Low urine pH affects the development of metabolic syndrome, associative with the increase of dyslipidemia and dysglycemia: Nationwide cross-sectional study (KNHANES 2013-2015) and a single-center retrospective cohort study
Source: PLoS One. 2018 Aug 24;13(8):e0202757. doi: 10.1371/journal.pone.0202757 (PMC6108487; doi:10.1371/journal.pone.0202757)
Supplement: S2 Table — Raised-TG: Adjusted for age, sex, eGFR, serum uric acid, and the values of another four MetS components; WC, BMI, HDL-cho, SBP, DBP, and FPG. Raised-FPG: Adjusted for age, sex, eGFR, serum uric acid, and the values of another four MetS components; WC, BMI, HDL-cho, SBP, DBP, and TG. (DOCX) [file pone.0202757.s002.docx]

**S2 Table. Additional cox regression analysis for new onset of Raised-TG and Raised-FPG, according to UpH values of participants without MetS at first checkup**

|  | **UpH≥7.0 (n=581)** | **UpH 6.0-6.5 (n=648)** | **UpH=5.5 (n=419)** | **UpH=5.0 (n=1,805)** | **P for trend** |
| --- | --- | --- | --- | --- | --- |
| **Raised-TG** | 1 (ref) | 0.908 (0.729-1.131) | 1.038 (0.817-1.319) | 1.251 (1.041-1.503)* | 0.001 |
| **Raised-FPG** | 1 (ref) | 1.126 (0.895-1.417) | 1.015 (0.779-1.322) | 1.354 (1.112-1.648) ** | 0.002 |

***p<0.001, **p<0.01, *p<0.05

Raised-TG : Adjusted for age, sex, eGFR, serum uric acid, and the values of another four MetS components; WC, BMI, HDL-cho, SBP, DBP, and FPG.

Raised-FPG : Adjusted for age, sex, eGFR, serum uric acid, and the values of another four MetS components; WC, BMI, HDL-cho, SBP, DBP, and TG.
